# The Transformation of Parents’ Perception of Education Involution Under the Background of “Double Reduction” Policy: The Mediating Role of Education Anxiety and Perception of Education Equity

**DOI:** 10.3389/fpsyg.2022.800039

**Published:** 2022-05-19

**Authors:** Shuo Yu, Jiansong Zheng, Zhibin Xu, Tao Zhang

**Affiliations:** ^1^Faculty of International Tourism and Management, City University of Macao, Macao, Macao SAR, China; ^2^Faculty of Humanities and Social Sciences, Macao Polytechnic University, Macao, Macao SAR, China; ^3^Institute of Analytical Psychology, City University of Macao, Macao, Macao SAR, China

**Keywords:** the “Double Reduction” policy, education anxiety, education equity, education involution, parents’ anxiety about children’s school admission, parents’ anxiety about children’s learning attitudes

## Abstract

The “Double Reduction” policy in China was introduced to reduce the academic burden of primary and secondary school students, ease their parents’ education anxiety, enhance education equity, and curb the phenomenon of education involution. A survey was conducted on 271 parents using the items of “Double Reduction” policy understanding level, subjective family social class, and education involution as well as the scales of anxiety about learning attitudes and school admission, as well as perception of education equity. The results showed that: (1) education anxiety acted as a significant mediator between parents’ understanding of the “Double Reduction” policy and their perception of education involution, with the full mediation of anxiety about school admission outperforming anxiety about learning attitudes and (2) the more parents understand about the “Double Reduction” policy, the higher their perceived education equity. In the initial stage of the “Double Reduction” policy implementation, a survey of parents’ education anxiety and their perception of the policy effect can gain an effective glimpse into the outcomes of the policy execution, especially in alleviating the education involution, observe the impact pathways that influence education involution, and provide adjustment and improvement for the “Double Reduction” operation in time.

## Introduction

On 24 July 2021, the General Office of the Communist Party of China Central Committee and the General Office of the State Council of the People’s Republic of China published *Opinions on Further Reducing the Burden of Homework and Off-Campus Training on Students in Compulsory Education* (hereinafter referred to as “Opinions”), namely the “Double Reduction” policy. China government requires schools to reduce students’ homework loading; off-campus training institutions cannot occupy national holidays, rest days, winter and summer holidays to organize subject-based training (The General Office of the Communist Party of China Central Committee and the General Office of the State Council of People’s Republic of China, 2021). The release of the “Opinions” aims to strengthen the role of the main school education, deepen the governance of off-campus training institutions, effectively relieve parents’ anxiety, resolutely prevent infringements on the interests of the public, build a safe educational environment, and promote the overall development and healthy growth of students.

The significant problem of current compulsory education is that the academic burden of primary and secondary school students is still too heavy, and there are still problems such as short-sightedness and utilitarianism. While there are problems such as students’ heavy homework burden, the problem of off-campus training, especially over-the-top training, remains severe. The unreasonable fees of some off-campus training programs have led to an excessive influx of capital. Some training institutions have broken the law such as “off-campus training institutions making off with the money” and “parents’ tuition fees being difficult to withdraw from off-campus training institutions.” These situations are a severe drain on parents’ financial and energy burdens. Worries about their children’s academic performance and future in higher education become a significant source of parents’ anxiety.

It seems that children’s learning pressure exists worldwide. In developed countries like the United States, parents tend to intervene throughout their children’s development, increasing their energy, time, and financial investment in all the aspects of early care, interest training, and extracurricular tuition (Ishizuka, 2019). Korea is also a very notable example. In Korea, the emergence of shadow education has led to significant inequality in Korean education (Choi and Park, 2016). Korea has made aggressive efforts to implement equality in education, but ultimately it has failed. The reason for the failure is that the policies enacted by the government, while relieving some of the pressure on students to some extent, have not made any changes to the status quo of high-intensity learning and the huge tutoring industry in Korea. Policies do not work to reduce and children’s learning anxiety. This problem also exists in developing countries, such as Turkey. Students, parents, and teachers in the countries have begun to recognize the importance of private tutoring for students’ success in university entrance exams, and many tuition agencies have emerged in society (Kurt and Gumus, 2021). Children seem to be under more and more pressure to learn across the world. China’s “Double Reduction” policy is designed precisely to reduce children’s stress to learn and the policy may provide opportunities for new learning paths. The policy may be able to provide an imitable idea for global education involution.

Education anxiety is a psychological anomaly caused by parents’ uncertainty about the process and results of their children’s education, due to various factors such as society, school, family, and students, which easily lead to restlessness, anxiety, sadness (Qian and Miao, 2021). Parental anxiety plays a negative role on the learning aspect of the child, and the child’s autonomy and motivation to learn will be greatly reduced (Wu et al., 2021). The dimensions of education anxiety can be divided into parents’ anxiety about children’s academic performance, parents’ anxiety about children’s learning attitudes (ACLA), and parents’ anxiety about children’s school admission (ACSA; Li, 2021). The introduction of the “Double Reduction” policy, which requires primary and secondary schools across China not to publish examination results before Chinese high school and college entrance examinations and to reduce the number of student examinations, has fundamentally eliminated parents’ anxiety about their children’s academic performance. As a necessary path for every student’s learning development, the admission of schools for further education is conducive to the optimization of education resources and has become the most pressing source of education anxiety for parents.

Family plays an important role for an individual in acquiring cultural capital (Bourdieu and Wacquant, 1992). Bourdieu’s theory considers cultural resources as a whole (Stempel, 2005). Those with higher family social classes have better access to cultural capital (Huang, 2019). In the theory, the dominant class uses cultural capital as an economic capital to distance itself from the subservient class. Priority of quality educational resources belongs to the dominant class (Stempel, 2005). One of the priorities of the “Double Reduction” policy process is to eliminate class rigidity, to break through class barriers, to enable students to grow naturally without class restrictions and to achieve educational tracking. In this case, the truly talented people can be selected to contribute themselves to the country’s scientific and technological development. The dominant class no longer has priority access to resources and may experience a class decline. An equitable educational environment gives the subservient class opportunities to make a class transcendence.

However, at the early stage of the implementation of the “Double Reduction” policy, parents’ short-sightedness and utilitarianism still existed. They distrusted the policy’s ability to reduce the burden of education and their fear of their children falling behind in school still existed (Zhang et al., 2021). Chinese parents prepare their children well for school before starting school in the early stages (Lau et al., 2011). The extent to which parents’ understanding of “Double Reduction” policy (UDRP) are likely to be associated with parents’ own education anxiety, including anxiety about learning attitudes and school admission. Based on this, this paper proposes the following hypothesis:

**Hypothesis 1 (H1):** The higher the level of parents’ understanding about the “Double Reduction” policy, the higher the parents’ education anxiety (i.e., parents’ anxiety about children’s learning attitudes and school admission).

Perception of education equity (PEE) refers to individuals’ perceptions and judgments of the degree of education equity in the current socio-cultural context (Zhang et al., 2019). Parents’ judgments of “fairness or unfairness” based on their own perception of the “proper state of education” and whether current education meets this standard, reflect parents’ subjective feelings and judgments about the fairness of education in the current environment (Zhu et al., 2018). Since 2018, a series of national measures have included adjusting the relationships between public and private education and between on-campus and off-campus education. The main thread running through them is to adhere to the public good nature of education and promote equity in education.

As a major initiative to uphold the principle of public welfare in education, the “Double Reduction” policy focuses on reducing the education expenses of each family while ensuring the development of education quality, thus achieving the effect of promoting education equity (Zhang, 2021). Parents’ perception of the “Double Reduction” policy in achieving education equity is important feedback on the implementation of this policy. Based on this, we propose the following hypothesis:

**Hypothesis 2 (H2):** The more parents know about the “Double Reduction” policy, the higher their perception of education equity.

The perception of education involution (PEI) refers to the degree to which individuals perceive the current phenomenon of education involution (Kyriakides, 2020). The current phenomenon of education involution in society is a product of shadow education. One family enrolls their children in one extracurricular class, while another family enrolls in two, or even a year or two ahead of time. The vicious competition among families leads to increased investment in education (Chen and Miao, 2021).

However, from the overall perspective, the number of people who can get into a high-quality high school or university is relatively stable. Students’ high test scores are obtained at the expense of the development of other abilities, leading to the phenomenon of high test scores and low ability from time to time (Liu and Neilson, 2011).

The continued proliferation of off-campus training is an endless drain on students and a bottomless demand for parents’ tuition output. The pressure on children to learn and parents’ anxiety about the future social status of their families are interwoven. Parents’ anxiety about their children’s learning attitudes and even school admission continues to rise (Ye, 2018). Education anxiety drives a continuous output of student energy and parents’ cash, and the phenomenon of involution of basic education deepens. Based on this, we propose the hypothesis that:

**Hypothesis 3 (H3):** Parents’ education anxiety, especially their anxiety about their children’s school admission, positively predicts parents’ perception of education involution.

The key to alleviating education involution is to promote education equity (Kyriakides, 2020). Drawing on the experience of German vocational education development, it is necessary to develop vocational education vigorously. The main reason why German vocational education is so popular is the equal distribution of incomes. The average annual salary of German university graduates is about 30,000 Euros, while the average annual salary of skilled workers reaches about 35,000 Euros. It is by achieving relatively equal benefits after education tracking, that we lay the foundations for avoiding the vicious competition of parents and students for certain education opportunities and achieve a balance in demand for talents in society (Sattin-Bajaj and Roda, 2020).

At this point, the problem of short-sightedness and utilitarianism is solved. Parents are able to allow their children to choose their own development direction based on their preferences, interests, and talents. Students are able to choose their learning paths from the perspective of all-round development. High-quality development of education can be achieved. The effective reduction of education involution can be also achieved. Therefore, we develop the hypothesis that:

**Hypothesis 4 (H4):** Parents’ perception of education equity negatively predicts their perception of education involution.

The important purpose of the “Double Reduction” work is to build a good education ecology, that is, to alleviate the phenomenon of education involution (Zhang, 2021). To grasp the “Double Reduction” policy reform scientifically and accurately, it is necessary to clarify the logical thinking of the “Double Reduction” policy reform and development. Through that, the authorities can get the real effect of the policy from the real feedback from the public and then constantly adjust the policy-related initiatives to get the optimal policy effect. Parents’ PEI is powerful feedback on the degree of implementation of the “Double Reduction” policy. This study aims to explore the extent to which the “Double Reduction” policy alleviates the current education involution and to provide real feedback on whether the “Double Reduction” policy has effectively reduced the phenomenon of education involution from the perspective of parents.

**Figure 1:**
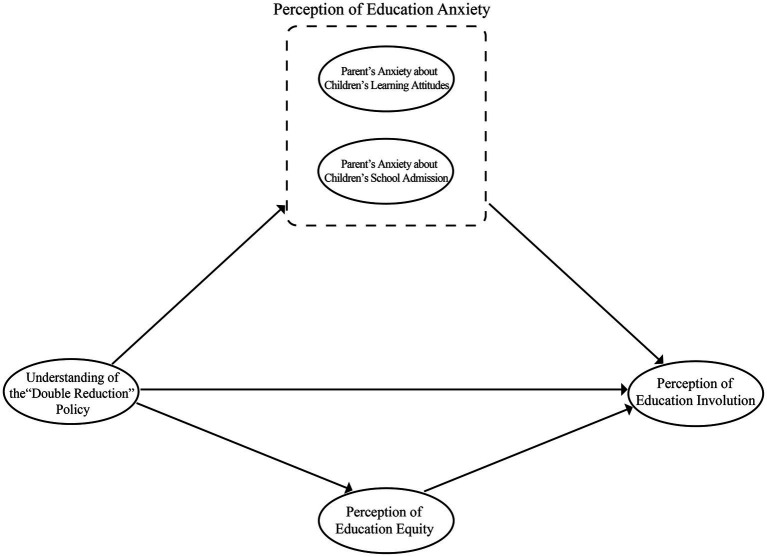
Theoretical framework.

Parents’ distrust of the national education policy has been a major obstacle to the implementation of the education policy (Zhang et al., 2021). The “Double Reduction” policy is an unprecedented policy to reduce the burden of education and aims to alleviate parents’ anxiety. The change of parents’ education anxiety affected by the “Double Reduction” policy is an important path to observe the degree of evolution of education involution under the policy implementation (Qian and Miao, 2021). At the same time, the promotion of education equity also facilitates the observation of the degree of transformation of education involution (Mao, 2021). Thus, parents’ education anxiety, including anxiety about their children’s learning attitudes, anxiety about their children’s school admission, and the PEE, may be powerful mediators of the “Double Reduction” policy’s impact on education involution (see Figure 1). As an exploratory study, the direction of influence of parents’ understanding of the “Double Reduction” policy on the PEI was not set (see Figure 1). Therefore, we develop the hypotheses that:

**Hypothesis 5a (H5a):** Parents’ understanding of the “Double Reduction” policy predicts parents’ perception of education involution.

**Hypothesis 5b (H5b):** Parents’ anxiety about their children’s learning attitudes mediates the relationship between parents’ understanding of the “Double Reduction” policy and their perception of education involution.

**Hypothesis 5c (H5c):** Parents’ anxiety about their children’s school admission mediates the relationship between parents’ understanding of the “Double Reduction” policy and their perception of education involution.

**Hypothesis 5d (H5d):** Parents’ perception of education equity mediates the relationship between parents’ understanding of the “Double Reduction” policy and their perception of education involution.

The “Double Reduction” policy is a great reform of China’s education burden reduction policy and has attracted widespread attention from society and academia since its implementation. The previous research on the “Double Reduction” policy is still at the level of theoretical analysis, exploring possible paths for the “Double Reduction” policy to help optimize the development of basic education ecology (Qian and Miao, 2021; Zhang, 2021; Zhang et al., 2021). According to a large-scale survey conducted by the Central Committee of the Communist Youth League of China on parents’ attitudes toward the “Double Reduction” policy, 87.0% of parents felt anxiety about their children’s education, and 72.7% of parents felt that their anxiety has eased after the implementation of the “Double Reduction” policy (Huang et al., 2021). However, none of the above works has empirically examined the ultimate goal of the “Double Reduction” policy (i.e., the path to eliminating education involution). Based on this, this present study investigates the perception of parents on education anxiety, education equity, and education involution after the implementation of the “Double Reduction” policy in order to fill the explanatory gap of the influencing pathways of the “Double Reduction” policy on education involution.

## Research Method

### Measure

The questionnaire collected the control variables including subjective family social class and children’s off-campus training participation, as shown in Table 1. Among them, higher subjective family social class scores represented individuals’ higher perception of their family’s social class, using a five-point scale; children’s participation in off-campus training was taken as “1: participated in training” and “0: did not participate in training.” Demographic variables were also collected, including gender, age, urban or rural household registration, household income, and education.

**Table 1:** Background of respondents.

Item	Frequencies	Percent (%)
**Gender**		
Male	94	34.7
Female	177	65.3
**Age**		
21–25	38	14.0
26–30	66	24.3
31–35	124	45.7
36–40	35	13.0
41–45	8	3.0
**Residence**		
Cities or towns	209	77.1
Countryside	62	22.8
**Education background**		
Primary school		
Junior high school	5	1.8
Senior high school	11	4.0
Three-year college	41	15.1
Undergraduate college	203	74.9
Master	10	3.6
Doctor	1	0.3
**Income (10,000)**		
Less than 12	47	17.3
12–70	215	79.3
Greater than 70	9	3.3
**Whether to participate in off-campus training**		
Yes	205	75.6
No	66	24.3
**Subjective family social class**		
Upper class	5	1.8
Upper middle class	47	17.3
Middle class	155	57.1
Lower middle class	61	22.5
Lower class	3	1.1

In this study, the two dimensions of parents’ ACLA and school admission were considered separately, and two parallel mediation models were constructed to test the model hypotheses shown in Figure 1. The study variables are the degree of parents’ understanding of the “Double Reduction” policy, education anxiety, PEE, and PEI.

Understanding of the “Double Reduction” Policy surveyed respondents’ degree of interpretation of *the Opinion*, as a single-item variable. We used the scale of parents’ anxiety about their children’s learning attitudes (five items) and school admission (seven items) by Li (2021) to measure parents’ education anxiety. We used the scale of parents’ PEE (three items) by Li (2021) to observe parents’ perception of current education equity. Based on the definition of PEI by Chen and Miao (2021), we described it as an item “It is education involution that extracurricular tutoring and excessive academic load can improve students’ academic performance, but it does not mean that students’ quality is improved, and it makes the competition for scores more intense and ineffective due to the stagnant acceptance rates.” Subjects were surveyed on their levels of agreement with the social condition.

The above variables were scored on a five-point scale with higher scores representing higher understanding of the “Double Reduction” policy, higher education anxiety, higher PEE, and higher PEI. The details of the questions are shown in Table 2.

**Table 2:** Reliability and validity of the constructs.

Research variables	Labels	Items	Mean	Std. Dev.	Skewness	Kurtosis	Cronbach’s alpha	Factor loadings	AVE	CR	VIF
Understanding of “Double Reduction” policy	UDRP	Are you aware of the recently enacted “Double Reduction” policy?	3.882	0.743	−0.757	2.039					
Parents’ anxiety about children’s learning attitudes	ACLA		3.812				0.814		0.57	0.869	
	ACLA1	In general, I am anxious about my child’s attitude toward learning.	3.672	0.922	−0.528	0.215		0.765			1.567
	ACLA2	I am worried about my child’s lack of self-motivation and lack of initiative in learning.	3.812	0.938	−0.784	0.639		0.776			1.830
	ACLA3	I am concerned that my child is afraid of learning.	3.768	0.967	−0.758	0.459		0.753			1.703
	ACLA4	I am worried about my child’s lack of interest in learning.	3.860	0.948	−0.741	0.193		0.709			1.584
	ACLA5	I am worried that my children will neglect their studies after they are addicted to playing with cell phones and computers.	3.948	0.901	−0.693	0.391		0.770			1.551
Parents’ anxiety about children’s school admission	ACSA		3.804				0.829		0.499	0.873	
	ACSA1	In general, I am anxious about my child’s school admission.	3.797	0.946	−0.667	0.185		0.786			1.997
	ACSA2	I would consider paying a higher school admission fee if it is difficult for my child to go to school.	3.720	0.971	−0.757	0.464		0.631			1.377
	ACSA3	I will worry about not living in the school district houses.	3.631	1.056	−0.662	0.019		0.702			1.633
	ACSA4	I am worried that my child will not go to the school of his expectation.	3.934	0.925	−0.859	0.685		0.700			1.783
	ACSA5	I am willing to spend time and effort to study the Ministry of Education’s admission policy.	3.845	0.838	−0.615	0.384		0.571			1.347
	ACSA6	I will ask around about the enrollment of each school.	3.904	0.855	−0.781	1.091		0.726			1.673
	ACSA7	I am torn about choosing a school for my children.	3.801	0.961	−0.702	0.251		0.801			2.124
Perception of education equity	PEE		3.749				0.784		0.696	0.873	
	PEE1	Every child has the same opportunity to access quality education resources.	3.753	0.944	−0.686	0.304		0.823			1.773
	PEE2	Every child can be treated equally in the education process.	3.775	0.921	−0.482	−0.147		0.801			1.479
	PEE3	Despite differences in family conditions, the understanding and skills that each child can learn are fair.	3.720	0.948	−0.569	0.014		0.877			1.757
Perception of education involution	PEI	In general, do you think that current education is involuted?	3.989	0.684	−0.365	−0.158					

### Date Collection

The target data collection objects of this study were parents of students of primary and secondary schools, and the data collection was completed in September 2021 at the website of collecting questionnaire https://www.wenjuan.com/list/. A convenience sampling method was used to collect 306 questionnaires, limiting the sample to those aged 21–45 years old, and screening samples with the number of children greater than or equal to 1 as the study population. Two hundred seventy-one valid questionnaires were yielded, with a valid questionnaire rate of 88.6%.

### Sample

The descriptive statistics of the subjective family social class, children’s off-campus training, and demographic information of the sample are shown in Table 1. The data showed that there were 177 female respondents, accounting for 65.3%; the age range of the subjects was [21, 45], with a mean value of about 31.2 and a variance of about 4.8. Two hundred five individuals, accounting for 75.6%, had their children involved in off-campus subject training. The number of individuals whose subjective family social class was in the middle class and above was 219, accounting for 80.7%. Females are more prone to anxiety and seem to be more sensitive than males (McLean and Anderson, 2009). There are possible age differences in psychological changes related to the “Double Reduction” policy. Considering that individual subjective family social class is a centralized generalization of information such as education, income, urban and rural household registration. The control variables were selected as gender, age, children’s participation in off-campus training, and subjective family social class.

## Data Analysis and Results

This study used partial least squares (PLS) estimation to model structural equations for two parallel mediated models and performed model testing. After controlling for variables, the results of the two models were compared. The reason for using PLS is the stronger applicability of PLS to the mediating role of the models (Lee et al., 2009), being able to extract maximized variance explained information for model testing (Haenlein and Kaplan, 2004), and model testing with non-normally distributed data having stronger robustness (Hair et al., 2012). We used SmartPLS 3.3 to run the PLS algorithm on 271 samples and took 5,000 bootstrapping samples to estimate the significance of the path coefficients (Dijkstra and Henseler, 2015).

### Reliability, Validity, and Correlation

In the statement of West et al. (1995), a skewness value higher than 2 and a kurtosis value higher than 7 are considered to jeopardize the univariate normality assumption of items. We then screen the individual responses to the 17 items to investigate skewness and kurtosis, whose values have ranges from −0.859 to −0.365 and from −0.158 to 2.039, with absolute mean values 0.671 and 0.449, respectively, as detailed in Table 2, indicating no severe violation of normality. Moreover, the PLS method applied here demonstrates more robustness with non-normal data (Hair et al., 2011) and is further facilitated by bootstrapping 5,000 samples in assessing the path coefficients’ significance according to the recommendation by Hair et al. (2012).

The reliability and validity of the study were moderately satisfied. Table 2 demonstrates the means, standard deviation, reliability, PLS factor loadings, average variance extracted (AVE), construct reliability (CR), and variance inflation factor (VIF) of the constructs. The results of the reliability test analysis showed that the Cronbach’s alpha coefficients of parents’ anxiety about their children’s learning attitudes, school admission, and PEE were all greater than 0.75. The data passed the reliability test. The results of the structural validity test showed that the PLS raw factor loadings were significantly greater than 0.7, except for the fifth item on parents’ anxiety about school admission. The data passed the structural validity test. The AVEs of parents’ anxiety about their children’s learning attitudes and PEE were greater than 0.5, while the AVE of parents’ school admission anxiety was 0.499, but the CR of parents’ anxiety about school admission anxiety was greater than 0.85, and the CRs of all other variables were also greater than 0.85, indicating that the reliability of the study was better (Fornell and Larcker, 1981). The VIFs for each question item were less than 3, indicating that there was no significant multicollinearity between the indicators of the study variables.

Fornell-Larcker criterion analysis was conducted on the research variables, including the degree of understanding of the “Double Reduction” policy, parents’ anxiety about their children’s learning attitudes, school admission, and PEE, education involution. The square root of AVE of parents’ anxiety about school admission was smaller than the Fornell-Larcker correlation coefficient between parents’ ACSA and parents’ ACLA, indicating the need for separate parallel mediation models to examine the mediating roles of the two dimensions of education anxiety, respectively. The square roots of AVEs for the remaining variables were all greater than the correlation coefficients between the cross variables, and the discriminant validity of both parallel mediation models was satisfied (Table 3).

**Table 3:** Latent variable correlations and square-root of AVE.

	Square-root of AVE	1	2	3	4
1. UDRP	-				
2. ACLA	0.755	0.190			
3. ACSA	0.707	0.323	0.746		
4. PEE	0.834	0.297	0.268	0.315	
5. PEI	-	0.121	0.327	0.371	0.026

UDRP represents parents’ understanding of the “Double Reduction” policy, ACLA presents parents’ anxiety about children’s learning attitudes, ACSA represents parents’ anxiety about children’s school admission, PEE represents perception of education equity, and PEI represents perception of education involution, similarly hereinafter.

### Results of PLS Analysis

**Figure 2:**
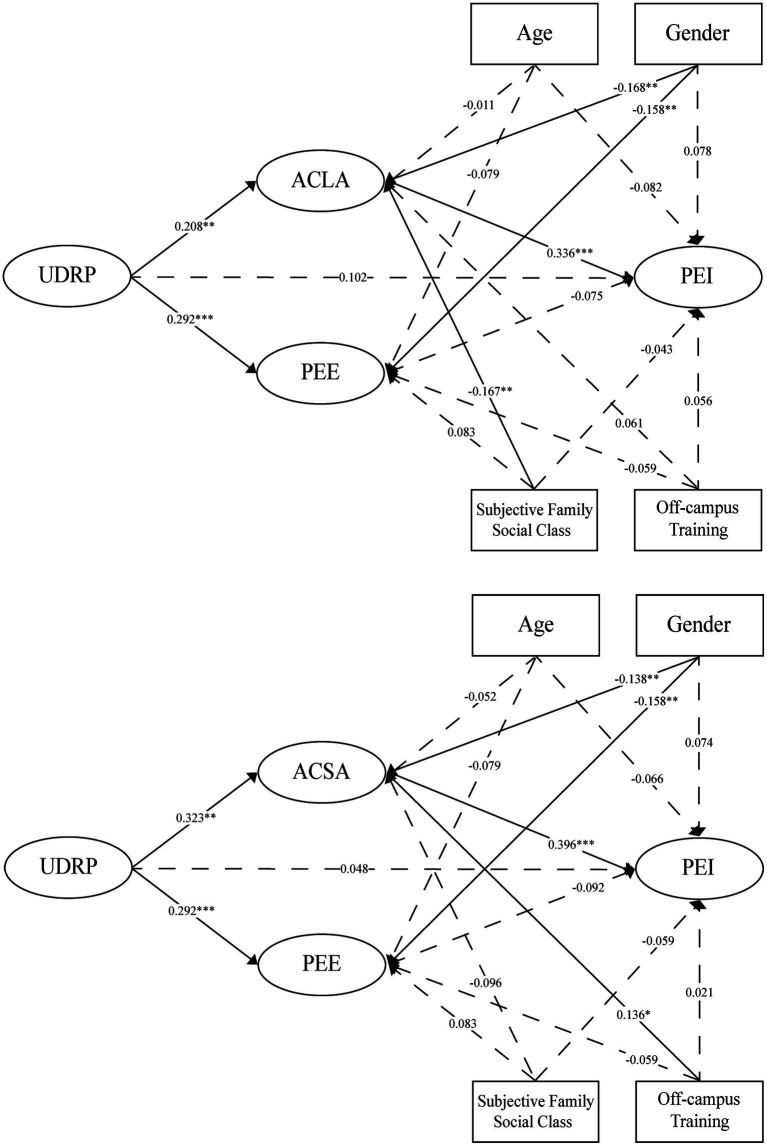
The results of PLS-SEM (above is model 1 and below is model 2). *N* = 271. *p < 0.05; **p < 0.01; ***p < 0.001.

Table 4 and Figure 2 reported the PLS estimates for the two parallel mediator models. The parallel mediation model with parents’ anxiety about their children’s learning attitudes as the mediating variable was assumed to be model 1. And, the parallel mediation model with parents’ anxiety about their children’s school admission as the mediator was assumed to be model 2. The test results of model 1 showed that parents’ understanding of the “Double Reduction” policy significantly positively predicted parents’ ACLA (*β* = 0.208, *p* = 0.003), parents’ understanding of the “Double Reduction” policy significantly positively predicted PEE (*β* = 0.292, *p* < 0.001), parents’ ACLA was a significant positive predictor of parents’ PEI (*β* = 0.336, *p* < 0.001), and parents’ PEE did not have a significant negative effect on education involution (*β* = −0.075, *p* = 0.301), parents’ understanding of the “Double Reduction” policy did not have a significant predictive effect on the PEI (*β* = 0.102, *p* = 0.104). Hypotheses H1, H2, and H3 were supported, and hypotheses H4 and H5a were not supported. The VIFs of the predictive relationships among the variables of model 1 ranged from 1.023 to 1.228, which was less than 3, indicating the absence of multicollinearity among the items of each latent variable (Hair et al., 2017). Model 1 fit was good (SRMR = 0.077, GoF = 0.296, *χ*^2^ = 208.1, NFI = 0.768).

**Table 4:** PLS path coefficient.

Path	Model 1			Model 2		
	Coefficient	*t*-value	VIF	Coefficient	*t*-value	VIF
UDRP→ACLA	0.208^**^	2.923	1.108			
UDRP→ACSA				0.323^***^	5.320	1.108
UDRP→PEE	0.292^***^	4.802	1.108	0.292^***^	4.871	1.108
UDRP→PEI	0.102	1.640	1.229	0.048	0.770	1.286
ACLA→PEI	0.336^***^	4.968	1.155			
ACSA→PEI				0.396^***^	6.307	1.245
PEE→PEI	−0.075	1.023	1.211	−0.092	1.314	1.220
Age→ACLA	−0.011	0.170				
Age→ACSA				−0.052	0.814	1.060
Age→PEE	−0.079	1.243	1.060	−0.079	1.236	1.060
Age→PEI	−0.082	1.311	1.067	−0.066	1.052	1.069
Gender→ACLA	−0.168^**^	2.809	1.023			
Gender→ACSA				−0.138^*^	2.314	1.023
Gender→PEE	−0.158^**^	2.678	1.023	−0.158^**^	2.651	1.023
Gender→PEI	0.078	1.322	1.071	0.074	1.218	1.064
Subjective family social class→ACLA	−0.167^**^	2.859	1.089			
Subjective family social class→ACSA				−0.096	1.739	1.089
Subjective family social class→PEE	0.083	1.351	1.089	0.083	1.375	1.089
Subjective family social class→PEI	−0.043	0.809	1.137	−0.059	1.097	1.114
Off-campus training→ACLA	0.061	0.977	1.063			
Off-campus training→ACSA				0.136^*^	2.132	1.063
Off-campus training→PEE	−0.059	0.967	1.063	−0.059	0.956	1.063
Off-campus training→PEI	0.056	0.950	1.073	0.021	0.363	1.095

*N* = 271.

* *p* < 0.05;

** *p* < 0.01;

*** *p* < 0.001.

The results of Model 2 showed that parents’ understanding of the “Double Reduction” policy significantly positively predicted parents’ anxiety about their children’s school admission (*β* = 0.323, *p* < 0.001), parents’ understanding of the “Double Reduction” policy significantly positively predicted PEE (*β* = 0.292, *p* < 0.001), and parents’ anxiety about their children’s admission school was a significant positive predictor of parents’ PEI (*β* = 0.396, *p* < 0.001). There was no significant negative effect of parents’ PEE on education involution (*β* = −0.092, *p* = 0.186), and no significant predictive effect of parents’ understanding of the “Double Reduction” policy on education involution (*β* = 0.048, *p* = 0.442). Hypotheses H1, H2, and H3 were supported again. H4 and H5a remained unsubstantiated. The VIFs of the predicted relationships among the variables of model 2 ranged from 1.023 to 1.286, which were less than 3, indicating the absence of multicollinearity among the items of each latent variable (Hair et al., 2017), compared to model 1, model 2 fitted better (SRMR = 0.081, GoF = 0.327, χ^2^ = 264.6, NFI = 0.772).

Hypothesis 5a was not directly supported, but the influencing path was significant with education anxiety as the mediating variable. Hypotheses 5b and 5c were supported, and hypothesis 5a was indirectly supported. Specifically, parents’ understanding of the “Double Reduction” policy influenced their anxiety about their children’s learning attitudes, which in turn influenced their PEI. Parents’ understanding of the “Double Reduction” policy influenced their anxiety about their children’s school admission, which in turn influenced their PEI. Both of them were fully mediated. The full mediation effect of model 2 was higher than that of model 1, and it can be inferred that the mediating effect of ACSA on parents’ PEI is greater than that about children’s learning attitudes (see Table 5). Hypothesis 5d was also not supported as the path of influence mediated by parents’ PEE was not significant.

**Table 5:** Mediation effect analysis.

Mediation path	Effect value	*t*-value	Significance C.I. (2.50%–97.5%)
**Model 1**			
UDRP→ACLA→PEI	0.070^*^	2.533	[0.014, 0.124]
UDRP→PEE→PEI	−0.022	0.972	[−0.066, 0.22]
**Model 2**			
UDRP→ACSA→PEI	0.128^***^	3.918	[0.064, 0.192]
UDRP→PEE→PEI	−0.027	0.022	[−0.07, 0.016]

*N* = 271. *p < 0.05; **p < 0.01; ***p < 0.001.

## Discussion and Conclusion

The purpose of the “Double Reduction” policy is to reduce the learning burden of students, relieve parents’ education anxiety, promote education equity, and alleviate the phenomenon of education involution. At the early stage of the policy implementation, it is helpful to understand the effect of the policy implementation and get real feedback from the first beneficiaries of the policy by understanding the parents’ education anxiety and perception of the effect of the “Double Reduction” policy. In this study, we measured parents’ education anxiety (i.e., ACLA and school admission), PEE, and PEI. This study constructed two parallel mediation models to explore the transformation of parents’ education anxiety and PEE in the context of implementation of the “Double Reduction” policy and to investigate the paths where education involution is affected by the “Double Reduction” policy. The results showed that (1) the more parents knew about the “Double Reduction” policy, the higher education anxiety they showed; (2) the more parents knew about the “Double Reduction” policy, the more they recognized the fairness of the current education situation; and (3) education anxiety mediated the association between parents understanding of the “Double Reduction” policy and education involution. Based on this, this paper proposed supporting measures for the “Double Reduction” policy in order to provide data support and theoretical suggestions for the implementation and promotion of the “Double Reduction” policy.

The ultimate goal of the “Double Reduction” policy is to alleviate the contemporary phenomenon of education involution. At the early stage of the policy implementation, the initial effect of the policy can be observed by scientifically measuring the perception of the first beneficiaries of the policy on education involution and exploring the influence paths of education involution, which is conducive to the steady progress and coordination of the “Double Reduction” work. This study found that the implementation of the “Double Reduction” policy did not directly cause parents to perceive the phenomenon of education involution, and only when parents’ education anxiety was incited did parents’ understanding of the “Double Reduction” policy significantly positively predict education involution. The mediating effect of anxiety about their children’s learning attitudes was weaker than that of anxiety about their children’s school admission. As a manifestation of outcome anxiety, contemporary parents’ anxiety about their children’s school admission is more urgent than the anxiety about their children’s learning attitudes. The PEI triggered by school admission anxiety is stronger.

Education anxiety, especially school admission anxiety, is closely linked to status anxiety as a form of cognitive anxiety. Education is an important way for individuals to cross the class line. High-quality school resources are geared to the status of the upper class, causing parents, especially middle class parents, to have the psychological need of “upper class envy.” While mediocre or even low quality school resources are geared to the status of the lower class, causing parents to have the psychological avoidance of “lower class fears,” including the fear of a bad reputation for their actual and expected status and the impact on their children’s future development expectations (Qian and Miao, 2021). In the face of education anxiety and status anxiety, parents’ energy and money, as well as children’s energy, are constantly involved in the off-campus training market. The understanding-doing gap in the current education process is caused by the tripartite dissonance between parents’ perception of the current involution of education, their understanding of the “Double Reduction” policy, and their children’s attachment in off-campus training.

The nature of the entry of off-campus training into the education market is an influx of capital into the education market, leading to a more severe inequity in education. According to Bourdieu’s theory of cultural capital reproduction, upper class families have higher cultural capital, and children of upper class families have easier access to cultural capital in the learning process compared to children of lower class families (Xie, 2021). Parents do not pay attention to the degree of education supply but rather pursue more and better education resources to improve their children’s competitive edge in learning, which essentially creates unequal education opportunities. The “Double Reduction” work is an unprecedented major initiative to regulate the off-campus training market in China, effectively cutting off the unequal access to education caused by off-campus training. Parents feel a higher level of education equity in the context of the “Double Reduction” policy implementation. The gradual withdrawal of capital from the field of basic education, the gradual emergence of justice in basic education, the transformation of the phenomenon of involution in basic education throughout China are the initial results of the implementation of the “Double Reduction” policy.

To achieve the sustainable development of basic education in China, the key still lies in establishing an education evaluation mechanism that can promote a virtuous cycle in the education ecosystem and truly realize the education requirement of “reducing the burden and improving the education quality” (Zhang et al., 2021). It is done by linking the vertical learning process and horizontal developmental elements of students. Then, this is achieved by promoting the assessment of outcomes, strengthening the assessment of processes, exploring value-added assessment and improving comprehensive assessment. The inherent social phenomena of credentialism, grade-ism, diploma-ism are gradually eliminated. The stereoscopic tracking and evaluation of students through big data tools promote the overall and coordinated development of students. At the same time, it is necessary to continue to improve and develop after-school services. As a project for the benefit of the people, the establishment of a sound mechanism for after-school services can effectively alleviate the anxiety of parents, especially urban parents, who find it difficult to pick up and drop off from work. The mechanism also can meet the individualized learning needs of students. Under strict regulation of off-campus subject training institutions, we will actively broaden the resource platform of famous teachers’ online classes in public schools and strive to let all non-tertiary school students throughout China enjoy high-quality learning resources nationwide.

There are still some shortcomings in this study. The degree of parents’ UDRP is a single-item, which is a weak measure. An extension of studies can consider using a questionnaire with correct answers based on the details of the “Double Reduction” policy to examine parents’ understanding of the “Double Reduction” policy and construct a scale to measure their understanding of the “Double Reduction” policy. PEI is a single-item entry that examines subjects’ agreement with the current education status of involution, which does not have complete validity and reliability. The next study can consider constructing a PEI scale to scientifically measure parents’ perception of the current education involution. In addition, the data used in this study are cross-sectional data, and it is unable to observe the policy effects of the “Double Reduction” work in the time cohort. The tracking surveys can be conducted to observe the psychological changes and the perceived policy effects during the process of “Double Reduction” work. Despite the limitations exist, this study has found that the evolutionary path of education involution on the implementation of the “Double Reduction” policy was mediated by parents’ education anxiety.

## Data Availability Statement

The original contributions presented in the study are included in the article/supplementary material, further inquiries can be directed to the corresponding author.

## Ethics Statement

Ethical review and approval was not required for the study on human participants in accordance with the local legislation and institutional requirements. Written informed consent for participation was not required for this study in accordance with the national legislation and the institutional requirements.

## Author Contributions

SY wrote the first draft of the manuscript. JZ designed the study and collected the survey data. ZX revised the first draft. SY and JZ conducted literature searches. SY, JZ, and ZX conducted the statistical analysis. TZ guided the writing of the first and revised draft of the paper and the application of statistical methods. All authors revised the revised draft, contributed to the article, and approved the submitted version.

## Funding

This work has been funded by the Research Project Fund of Macao Polytechnic University (RP/ESCHS-04/2020) and the Southern Marine Science and Engineering Guangdong Laboratory (Zhuhai) Annual Project (SML2020SP002).

## Conflict of Interest

The authors declare that the research was conducted in the absence of any commercial or financial relationships that could be construed as a potential conflict of interest.

## Publisher’s Note

All claims expressed in this article are solely those of the authors and do not necessarily represent those of their affiliated organizations, or those of the publisher, the editors and the reviewers. Any product that may be evaluated in this article, or claim that may be made by its manufacturer, is not guaranteed or endorsed by the publisher.
